# Effect of gestational oily fish intake on the risk of allergy in children may be influenced by *FADS1/2*, *ELOVL5* expression and DNA methylation

**DOI:** 10.1186/s12263-019-0644-8

**Published:** 2019-06-18

**Authors:** Purevsuren Losol, Faisal I. Rezwan, Veeresh K. Patil, Carina Venter, Susan Ewart, Hongmei Zhang, S. Hasan Arshad, Wilfried Karmaus, John W. Holloway

**Affiliations:** 1 Human Development and Health, Faculty of Medicine, University of Southampton, Southampton, UK; 2 Department of Molecular Biology and Genetics, School of Biomedicine, Mongolian National University of Medical Sciences, Ulaanbaatar, Mongolia; 3 The David Hide Asthma and Allergy Research Centre, Isle of Wight, UK; 4 Clinical and Experimental Sciences, Faculty of Medicine, University of Southampton, Southampton, UK; 5 Department of Large Animal Clinical Sciences, Michigan State University, East Lansing, MI USA; 6 Division of Epidemiology, Biostatistics, and Environmental Health, School of Public Health, University of Memphis, Memphis, TN USA

**Keywords:** Allergy, FADS, ELOVL, Fish intake, DNA methylation, Pregnancy

## Abstract

**Background:**

Evidence suggests that prenatal exposure to *n-3* long-chain polyunsaturated fatty acids (LCPUFA) reduces the incidence of allergic disease in children. LCPUFAs are produced from dietary precursors catalyzed by desaturases and elongases encoded by the *FADS1/2* and *ELOVL5* genes. DNA methylation regulates gene activity and fatty acid supplementation could alter DNA methylation (DNA-M) at these genes. We investigated whether DNA-M and expression of the *FADS1/2* and *ELOVL5* genes were associated with allergy in children and gestational fish intake. We studied 170 participants from the Isle of Wight 3rd Generation Cohort, UK. Phenotype data and exposure was assessed by questionnaires. Genome-wide DNA-M in cord blood samples was quantified using the Illumina Infinium HumanMethylation450 and EPIC Beadchips. Five SNPs (single-nucleotide polymorphisms) in the *FADS* gene cluster and one SNP in *ELOVL5* were genotyped in offspring. *FADS* gene expression in offspring cord blood was determined.

**Results:**

Gestational fish intake was significantly associated with increased methylation of cg12517394 (*P* = 0.049), which positively correlated with *FADS1* mRNA levels (*P* = 0.021). *ELOVL5* rs2397142 was significantly associated with eczema (*P* = 0.011) and methylation at cg11748354 and cg24524396 (*P* < 0.001 and *P* = 0.036, respectively). Gestational fish intake was strongly associated with elevated DNA-M at cg11748354 and cg24524396 (*P* = 0.029 and *P* = 0.002, respectively) and reduced *ELOVL5* mRNA expression (*P* = 0.028).

**Conclusion:**

The association between induced *FADS1/2* and *ELOVL5* DNA-M and reduced gene expression due to gestational fish intake provide a mechanistic explanation of the previously observed association between maternal LCPUFA intake and allergy development in early childhood.

**Electronic supplementary material:**

The online version of this article (10.1186/s12263-019-0644-8) contains supplementary material, which is available to authorized users.

## Background

Maternal diet during pregnancy is a potentially important determinant of intrauterine development and linked to fetal physiologic adaptations and early immune system programming [1]. To maintain fetal development, nutrients are transported to the fetus across the placenta [2]. Exposure to omega-6 (*n-6*) and omega-3 (*n-3*) polyunsaturated fatty acids (PUFAs) during pregnancy has been found to be associated with allergic outcomes in infants or children [3]. Maternal PUFA supplementation during pregnancy has been reported to reduce childhood asthma at age 16 [4] and to reduce the absolute risk of persistent wheeze or asthma and airway inflammation in offspring at 36 months after birth [5]. Oily fish consumption, a major source of *n-3* PUFAs, contains higher amount of eicosapentaenoic acid (EPA) and docosahexaenoic acid (DHA) which has been shown to be protective against allergic diseases [6]. Fish intake has also been demonstrated to diminish the risk of asthma at 18 months [7]. In contrast, maternal shellfish consumption during the first trimester has been shown to increase the risk of wheezing, eczema and food allergy, while fatty fish consumption has been associated with increased risk of eczema in offspring [8, 9]. However, a recent large prospective study could not substantiate the previously observed beneficial association between fish and seafood consumption in pregnancy and development of asthma and allergic rhinitis symptoms in children up to 8 years of age [10]. In contrast, a meta-analysis of six studies revealed an association between fish oil supplementation during pregnancy and reduced risk of sensitization to food allergens at first year [11].

Long-chain PUFAs (LCPUFAs), produced from their dietary precursors (omega-3 and omega-6), are catalyzed by desaturases and elongases which are encoded by the *FADS1/2* and *ELOVL2/5* genes. A number of studies have found an association between *FADS* gene variants and immune-related outcomes. For example, maternal *FADS* genetic variation, through a higher infant supply of LCPUFA, has been found to be associated with a decrease in the production of IL-5, IL-10, and IL-17 in the infant [12–16]. Carriers of the minor alleles of *FADS* genetic variants and their respective haplotypes have been demonstrated to have lower levels of desaturase products and a lower prevalence of allergic rhinitis and atopic eczema [16]. An observational study showed that the *FADS* SNP rs3834458 was significantly associated with serum LCPUFA levels, with individuals homozygous for the del/del variant shown to have a decreased level of arachidonic acid (AA) and increased alpha-linolenic acid (ALA) to DHA ratio in high fish-eating mothers [17]. Previously, it has been reported that carriers of the minor alleles of *FADS* SNPs, including rs3834458, tend to have a lower blood composition of LCPUFA, particularly AA [18]. Although the evidence is not conclusive, it is conceivable that minor alleles of *FADS* genes produce a lower proportion of desaturase products and therefore less AA, reducing the risk of asthma.

Increased DNA methylation (DNA-M) at *FADS* promoter regions was associated with lower gene expression levels in minor homozygote carriers [19], and differences in DNA-M associated with the development of asthma during childhood has been reported [20, 21]. There are suggestions that DNA-M may regulate *FADS* activity [22] and that fatty acid supplementation can induce altered methylation of specific CpG loci in *FADS*2 and *ELOVL5* [23]. A murine study reported differing levels of *FADS2* promoter methylation in the liver tissue from offspring exposed to linoleic acid during gestation [24]. Recently, allele-specific methylation was reported between rs174537 and DNA methylation in *FADS* region in leukocyte and CD4+ cells [25].

Given these interrelations between maternal diet, genotype, offspring DNA-M, and asthma, we hypothesized that maternal fish intake may modulate offspring epigenetic programming, regulating fatty acid desaturase and elongase activities, and that this may modulate later health outcomes, in particular childhood wheeze and eczema.

## Results

**Fig. 1:**
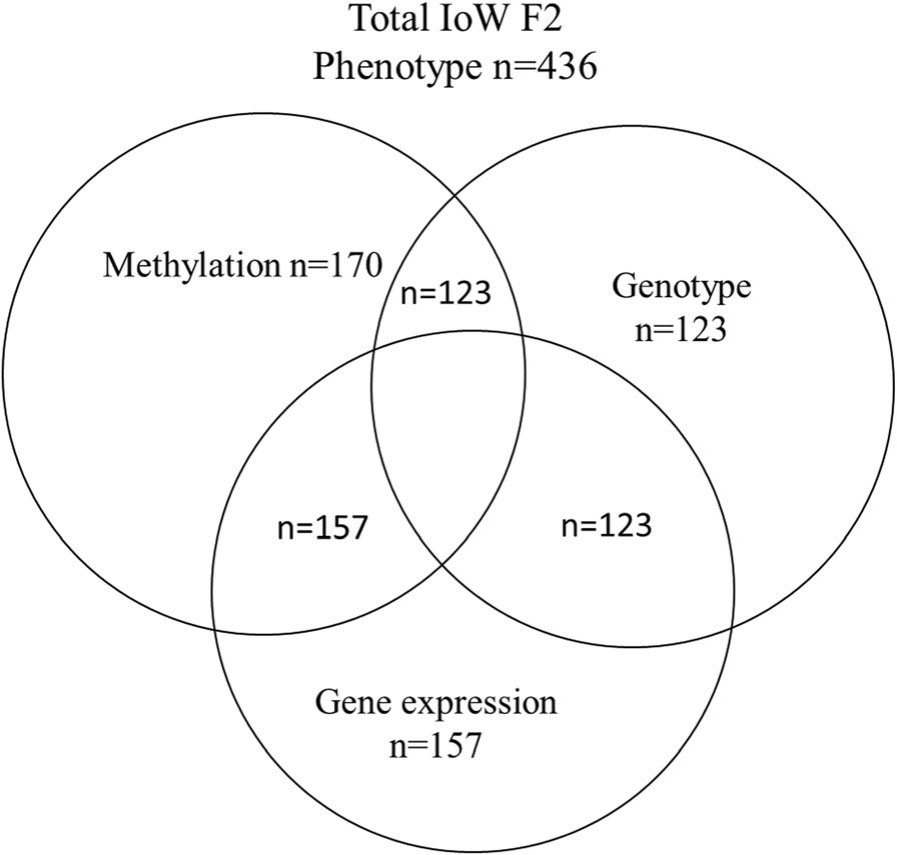
Venn diagram of participants by different subgroups

The descriptive characteristics of the cohort are presented in Table 1. There were no substantial differences in the prevalence of maternal smoking during pregnancy, maternal history of asthma, maternal history of eczema, maternal socioeconomic status, child eczema, child wheeze, and maternal oily fish intake between the total cohort and those who were randomly selected for the DNA-methylation analysis (Table 1). For each *FADS* and *ELOVL5* association analysis, 170 methylation-phenotype pairs, 157 methylation-gene expression pairs and phenotype-gene expression pairs, 123 methylation-genotype, phenotype-genotype, and gene expression-genotype pairs were available (Fig. 1).

**Table 1:** Characteristics of subjects with available methylation data compared to the participants of the total

	Total participants *n* = 436 (%)	Participants with DNA-M data *n* = 170 (%)	*P* value
Maternal smoking during pregnancy			
Yes	146 (36.6)	61 (37.0)	> 0.999
No	253 (63.4)	104 (63.0)	
Maternal history of asthma			
Yes	64 (18.4)	27 (18.2)	> 0.999
No	284 (81.6)	121 (81.8)	
Missing	88	22	
Maternal history of eczema			
Yes	143 (41.6)	65 (40.4)	0.846
No	201 (58.4)	96 (59.6)	
NA	92	9	
Child eczema			
Yes	58 (15.8)	29 (18.7)	0.442
No	308 (84.2)	126 (81.3)	
Missing	70	15	
Child wheeze			
Yes	67 (18.4)	32 (20.6)	0.625
No	298 (81.6)	123 (79.4)	
Missing	71	15	
Maternal fish intake			
Never	159 (61.9)	70 (61.4)	0.801
1–2 or less per month	74 (28.8)	32 (28.1)	
1–3 per week	21 (8.2)	11 (9.6)	
4 or more times per week	2 (0.8)	0 (0)	
Uncertain	1 (0.4)	1 (0.9)	
Missing	179	52	
Maternal socioeconomic status			
Below low	77 (19.3)	25 (15.1)	0.273
Low	97 (24.3)	54 (32.5)	
Medium Low	114 (28.5)	48 (28.3)	
Medium	61 (15.3)	24 (14.5)	
High	51 (12.8)	16 (9.6)	
NA	36	3	

Data are *n* (%), unless otherwise indicated. Difference between groups was assessed using Yates’ continuity correction

*DNA-M* DNA methylation, *NA* not applicable

Three *FADS1* and *FADS2* SNPs (rs174537, rs174556, rs174575) and one *ELOVL5* SNP (rs2397142) were genotyped and used for further analysis as these were not in linkage disequilibrium (*r*^2^ ≤ 0.80). Associations of these SNPs with the prevalence of wheeze and eczema were assessed as shown in Table 2. Among these, the *ELOVL5* variant rs2397142 was associated with eczema (*P* = 0.011). The eczema prevalence was higher in minor allele carriers than in homozygous major allele carriers.

**Table 2:** Prevalence of wheeze and eczema in offspring stratified by genotype

Gene	SNPs	Genotypes	Wheeze ever *n* (%)	*P* value	Eczema ever *n* (%)	*P* value
*FADS1*	rs174537	GG	11 (21.6)	> 0.999	10 (19.6)	0.849
		GT	11 (22.0)		12 (23.5)	
		TT	3 (25.0)		2 (16.7)	
*FADS1*	rs174556	CC	11 (20.0)	0.669	11 (20.0)	0.939
		CT	11 (24.4)		11 (23.9)	
		TT	1 (12.5)		2 (25.0)	
*FADS2*	rs174575	CC	12 (19.4)	0.614	12 (19.4)	0.939
		CG	10 (23.8)		10 (23.3)	
		GG	3 (33.3)		2 (22.2)	
*ELOVL5*	rs2397142	CC	9 (20.0)	0.569	4 (8.9)	0.011
		CG	14 (25.9)		18 (32.7)	
		GG	2 (14.3)		2 (14.3)	

Data are *n* (%). Chi-square test was applied with Yates’ correction

A total of 39 CpG sites spanning the genomic region of the *FADS* cluster and 27 CpG sites in *ELOVL5* were analyzed for association with wheeze and eczema. Methylation levels of cg00786201 and cg25448062 in the *FADS* cluster showed an association with eczema, and methylation at cg01400685 was found to be associated with wheeze (*P* < 0.05, respectively; Additional file 1). Also, methylation levels of cg18564099 in *ELOVL5* showed an association with eczema (*P* = 0.046; Additional file 1).

Maternal oily fish intake was correlated with significantly increased DNA-M level at cg12517394 in *FADS* (*P* = 0.049, Table 3).

**Table 3:** Association of maternal fish intake with cord blood *FADS* DNA methylation

CpG	Oily fish intake (28 weeks)		*P* value
	No (*n* = 103)	Yes (*n* = 11)	
cg00614641	0.088 ± 0.013	0.079 ± 0.010	0.022
cg07999042	0.880 ± 0.013	0.870 ± 0.020	0.033
cg12517394	0.037 ± 0.007	0.040 ± 0.005	0.049

Data are mean (SD). *P* values calculated using the Mann-Whitney *U* test

In addition, maternal oily fish intake was strongly associated with elevated DNA-M at cg11748354 and cg24524396 in *ELOVL5* (*P* = 0.029 and *P* = 0.002, respectively, Table 4) and reduced *ELOVL5* mRNA expression (*P* = 0.028, Table 5).

**Table 4:** Association of maternal fish intake with cord blood *ELOVL5* DNA methylation

CpG	Oily fish intake (28 weeks)		*P* value
	No (*n* = 103)	Yes (*n* = 11)	
cg10410213	0.608 ± 0.025	0.079 ± 0.020	0.010
cg11748354	0.146 ± 0.045	0.191 ± 0.073	0.029
cg24524396	0.093 ± 0.011	0.106 ± 0.012	0.002

Data are mean (SD). *P* values calculated using the Mann-Whitney *U* test

**Table 5:** Gestational oily fish intake on offspring gene expression

	Oily fish intake (28 weeks)		*P* value
	No (*n* = 81)	Yes (*n* = 9)	
*FADS1* mRNA	6.77 ± 0.42	6.68 ± 0.52	0.691
*FADS2* mRNA	12.13 ± 0.80	12.66 ± 0.90	0.090
Intergenic mRNA	8.57 ± 0.59	8.95 ± 0.62	0.104
*ELOVL5* mRNA	8.47 ± 0.43	7.93 ± 0.63	0.028

Data are mean (SD). *P* values calculated using the Mann-Whitney *U* test

A genotype-dependent interaction was identified between *ELOVL5* SNPs and CpG sites (cg11748354 × rs2397142 and cg24524396 × rs2397142 (*P* < 0.001 and *P* = 0.036, respectively; Additional file 2). When the correlation between methylation levels and gene expression levels in cord blood were tested, DNA-M at cg12517394 was significantly correlated with *FADS1* gene expression levels (*P* = 0.021, Additional file 3). *FADS1* gene expression levels were significantly different according to the *FADS* SNPs (*P* < 0.05, Additional file 4), but not in *FADS2* and *ELOVL5*.

## Discussion

This study investigated the effects of oily fish intake during the third trimester of pregnancy on the allergic outcomes of the infants by using a subset of umbilical cord blood samples from Isle of Wight (IoW) F_2_ generation. This is the first study to explore associations between *FADS* and *ELOVL* SNPs, gene expression, DNA methylation, child allergy, and gestational oily fish intake in the same cohort. Maternal fish intake could increase mean maternal circulating omega-3 levels and increased cord blood fatty acids that potentially prevent inflammation [26].

Our first major finding was a significantly increased cord blood methylation of cg12517394 in the *FADS* cluster and DNA methylation at *ELOVL5*, cg11748354, and cg24524396 with gestational oily fish intake, whereas oily fish intake during pregnancy reduced *ELOVL5* mRNA expression in the cord blood. In previous studies, supplementation with *n*-3 LCPUFA was shown to increase methylation levels at CpGs in *FADS2* and *ELOVL5*, which were negatively associated with the level of the *FADS2* and *ELOVL5* transcripts in non-atopic adults consuming *n*-3 LCPUFA for 8–12 weeks [23]. In contrast, it was reported that maternal fish oil supplementation during the second half of pregnancy had small or no effects on DNA-M of infants [27, 28]. In vivo, feeding rats a fish oil-enriched diet for 9 weeks induced lower *FADS2* mRNA expression and increased methylation of specific CpG loci in *FADS2* [29]. Together, these findings suggest that methylation at these loci directly regulates *FADS* and *ELOVL5* transcription, and *n*-3 LCPUFA intake can induce changes in methylation levels in specific genes.

Our second major finding was a genotype-dependent methylation of cg11748354 and cg24524396 related to *ELOVL5* rs2397142. A previous meta-analysis in liver methylation data, a highly relevant tissue for PUFA metabolism, identified a strong effect of *FADS* rs174537 on the methylation status of one or more critical CpG sites in the *FADS* gene cluster [19]. The most significant association was observed with cg27386326 (*p* = 2.69 × 10^−29^) and four other sites, including cg16213375, cg10515671, cg03805684, and cg19610905 [22]. We did not observe genotype-dependent methylation of *FADS1*. However, we identified higher gene expression levels for *FADS1* in the cord blood with positive correlation with methylation at cg12517394 that are likely to be influenced by *FADS* genetic variants.

Finally, rs2397142 in *ELOVL5* showed significant association with eczema in this study. The frequency of GG genotype of this polymorphism was higher in children with eczema. In a previous study, children from mothers with low fatty fish consumption during pregnancy had higher risk of eczema [8]. Furthermore, children with atopic eczema had lower *ELOVL5* mRNA levels in their blood when compared to healthy controls [30]. These findings indicate that maternal lower fatty acid levels during pregnancy directly associated with decreased *ELOVL5* mRNA expression in children and increase the risk of eczema, especially in children carrying GG genotype of rs2397142.

Our study had some limitations. Data on DNA-M and gene expression were only available from the cord blood and not from the liver samples, the major site of fatty acid metabolism. In addition, only proxy measures of maternal LCPUFA intake in the form of self-reported oily fish intake were available. We were unable to determine the ration of omega-6 to omega-3 in this cohort. This has previously been indicated as a possible risk factor for the development of atopic dermatitis and allergic rhinitis [31] and should be addressed in future studies. Nonetheless, the SNP-DNA-M gene expression associations observed largely support previous observations. Finally, this study was underpowered to investigate weaker links between maternal oily fish intake and clinical outcomes in children; however, there was a trend for a lower incidence of early life wheeze in the group whose mothers consumed oily fish compared with those who did not.

## Conclusions

We report that infants of mothers supplemented with higher oily fish intake during the last trimester of pregnancy had greater methylation levels at *FADS* gene cluster and *ELOVL5* and lower levels of *ELOVL5* mRNA expression. These methylation and gene expression changes were modulated by *FADS* and *ELOVL* gene variants. In conclusion, the association between induced *FADS* and *ELOVL* DNA methylation and reduced gene expression due to gestational fish intake gives a mechanistic explanation of previously observed associations between maternal LCPUFA intake and allergy development in early childhood.

## Methods

### Study population

Children born on the Isle of Wight (IoW) between 1989 and 1990 (*n* = 1536) were recruited to prospectively study the natural history of asthma, allergy, and obesity. Informed consent was obtained from the parents (1st generation, IoW F_0_), and after exclusion, 1456 participants were recruited (2nd generation, IoW F_1_) as the IoW birth cohort [32]. The recruitment of newborns for the IoW 3rd Generation (IoW F_2_) study started in April 2010, and the samples used in this study are from infants born between April 2010 and May 2018. In total, 436 newborns have been recruited such that at least one of their parents is in the IoW F_1_ [33]. In this study, we have used cord blood samples from a subset of IoW F_2_ generation.

### Clinical data collection

In the F_2_ generation, maternal history of asthma and smoking during pregnancy was ascertained at birth. Early wheezing was defined if symptoms of “wheeze” were reported by parents to occur between cold/infections at least once at either the 3-, 6-, or 12-month follow-up after birth. Childhood eczema information was also collected at 3, 6, and 12 months and defined as chronic or chronically relapsing, itchy dermatitis lasting more than six weeks with characteristic morphology and distribution [34, 35].

### Assessment of fish and shellfish intake

Participant’s mothers were asked to fill out a validated food frequency questionnaire that inquired about their usual consumption, before pregnancy, during 24th and 28th week of gestation [36]. White fish, shellfish, and oily fish consumption was evaluated with women reporting their frequency of consumption on a 5-point scale: “never,” “rarely (1–2 or less per month),” “occasionally (1–3 per week),” “4 or more times per week,” and “uncertain.” Mackerel, salmon, sardines, pilchards, herring, kipper, whitebait, trout, crab, and fresh tuna were classified as oily fishes. We considered oily fish intake only in the current study because of its high omega-3 contents.

### SNP selection for FADS1, FADS2, and ELOVL5 genes

Five candidate SNPs—*FADS1* (Entrez Gene 3992) rs174537, rs174545, and rs174556; intergenic between *FADS1-FADS2* (Entrez Gene 9415) rs3834458; and *FADS2* rs174575 and *ELOVL5* rs2397142—were selected based on evidence of association with LCPUFA proportions in human plasma, tissues, and milk [37], or because the SNPs had been suggested to play an important role in regulation of FADS1/2 activity because of their location near potential regulatory regions [16]. Three of the five SNPs (rs174537, rs174545, and rs3834458) are from the same haplotype block (*r*^2^ > 0.80). Therefore, in our analysis, we have only used four unrelated SNPs: rs174537, rs174556, rs174575, and rs2397142. For the IoW F_2,_ genotyping was conducted using cord blood DNA samples (*n* = 123) with the genome-wide Infinium OmniExpressExome-8 Kit, and all six SNPs were imputed using the 1000 genome reference panel [38].

### DNA methylation

DNA was extracted from the cord blood samples of IoW F_2_ over six batches, using a standard salting out procedure [39]. The DNA concentration was determined by PicoGreen quantitation. One microgram of DNA was bisulfite-treated for cytosine to thymine conversion using the EZ 96-DNA methylation kit (Zymo Research, Irvine, CA, USA), following the manufacturer’s standard protocol. Genome-wide DNA-M was assessed in 130 samples using the Illumina Infinium HumanMethylation450 BeadChip (Illumina, Inc., San Diego, CA, USA) and using the Illumina Infinium MethylationEPIC Beadchip array for 63 samples. Arrays were processed using a standard protocol as previously described [40], with multiple identical control samples assigned to each bisulphite conversion batch to assess assay variability, and samples were randomly distributed on microarrays to control against batch effects. The degree of DNA methylation at each CpG site was recorded as beta value, ranging from 0 (no methylation) to 1 (completely methylated). One sample was excluded due to maternal blood contamination. The CPACOR pipeline [41] was used to pre-process the methylation 450k and EPIC data for quality control, and batch correction was done using ComBat [42]. In the final analysis, 170 samples were used with complete methylation information. The methylation levels of 39 CpG sites spanning the genomic region of the *FADS* cluster and 27 CpGs of the *ELOVL5* were selected.

### Gene expression

*FADS* and *ELOVL5* gene expression in the cord blood (*n* = 157) was determined by total RNA extracted from the cord blood using SurePrint G3 Human Gene Expression Microarrays (GeneSpring Technology 39,494) [43]. Microarray data was processed using *limma* [43] in the R statistical computing environment, and background correction was performed using normal-exponential convolution (normexp) function [44]. Data was converted to log2-transformed data for further analysis. Filtering was performed to remove low expressed probes that are close to the background level. Negative control probes were also removed from the data.

### Statistical analysis

To assess whether our analytic sample (170 DNA samples) was representative of the total cohort available (*n* = 436), we compared the characteristics of the two subsets using chi-squared tests. Distribution and prevalence of wheeze and eczema according to genotype were compared using the *χ*^2^ test with Yates’ continuity correction. The genotype frequency of candidate SNPs was examined for a significant departure from the Hardy-Weinberg equilibrium using a *χ*^2^ test. The methylation levels of the *FADS* cluster and *ELOVL5* were tested for association with wheeze and eczema up to 1 year using Mann-Whitney *U* tests to compare independent samples. SNP genotype-dependent methylation and genotype-dependent gene expression were analyzed using the Kruskal-Wallis non-parametric test to determine the difference between three genotype groups on continuous variables. A Spearman correlation test was used to analyze the relationship between DNA methylation levels and gene expression. Comparison of DNA-M and gene expression levels between oily fish intake status at 28 gestational weeks were tested by the non-parametric two-sample Mann-Whitney *U* test. Bonferroni correction was not undertaken as these were a priori hypothesis instead of a random set of CpGs and genotypes. Under this situation, multiple testing adjustments may not be necessary as suggested by Rothman [45].

All statistical analyses were performed using IBM SPSS Statistics, Version 21.0 (IBM Corp., Armonk, NY, USA), except for preprocessing of DNA methylation data, which was done using R statistical package [41]. The *P* values of < 0.05 were considered to indicate significance.

## Additional files

Additional file 1: Association of wheeze and eczema with cord blood DNA methylation. (XLSX 9 kb)

Additional file 2: Genotype-dependent methylation. (XLSX 9 kb)

Additional file 3: Change in relative mRNA expression compared to change in the methylation status of FADS1 methylation cg12517394. (XLSX 8 kb)

Additional file 4: Gene expression according to the *FADS* genotypes. (XLSX 9 kb)

## Data Availability

The datasets used and/or analyzed during the current study are available from the corresponding author on reasonable request.

## Abbreviations

AA: Arachidonic acid
ALA: α-Linolenic acid
DHA: Docosahexaenoic acid
DNA-M: DNA methylation
ELOVL: Fatty acid elongase
EPA: Eicosapentaenoic acid
FADS: Fatty acid desaturase
IL: Interleukin
IoW: Isle of Wight
LCPUFA: Long-chain polyunsaturated fatty acids
mRNA: Messenger RNA
SNP: Single-nucleotide polymorphism
